# Effect of the COVID-19 Outbreak on the Incidence of Other Respiratory and Gastrointestinal Infections in Children in Thai Binh, Vietnam in 2020

**DOI:** 10.1007/s44197-022-00037-5

**Published:** 2022-04-10

**Authors:** Quoc Tien Nguyen, Thi Loi Dao, Thi Dung Pham, Trong Kiem Tran, Van Thuan Hoang, Philippe Gautret

**Affiliations:** 1 Thai Binh University of Medicine and Pharmacy, Thai Binh, Vietnam; 2 Thai Binh Pediatric Hospital, Thai Binh, Vietnam; 3 Aix Marseille Univ, IRD, AP-HM, SSA, VITROME, Marseille, France; 4 Institut Hospitalo-Universitaire Méditerranée Infection, 19-21 Boulevard Jean Moulin, 13385 Marseille Cedex 05, France

**Keywords:** SARS-CoV-2, COVID-19, Respiratory tract infection, Gastrointestinal infection, Children

## Abstract

**Introduction:**

To evaluate the impact of COVID-19 mitigation measures on the total number of consultations for respiratory and gastrointestinal infections among children under 16 years in Thai Binh Pediatric Hospital, Vietnam during the year 2020.

**Methods:**

A retrospective study was carried out to review consecutive consultations occurring in children admitted from January 01, 2016 to December 31, 2020. All medical records were collected from the central numeric database of the hospital. Diagnoses were documented according to the International Classification of Diseases 10 criteria.

**Results:**

436,276 children consulted at the outpatient department during the period of study. A gradual increase in the total number of outpatients was observed from 2016 to 2019, including those consulting for respiratory and gastrointestinal infections. However, the total number of outpatients and the numbers of those consulting for respiratory and gastrointestinal infections dramatically decreased in 2020. A significant decrease of respiratory infections relative proportion was observed in 2020 when compared to 2016–2019 (*p* < 0.0001). By contrast, the relative proportion of gastrointestinal infections did not significantly vary (*p* = 0.91). The proportion of outpatients aged under 5 years was significantly lower in 2020 compared to previous years (*p* < 0.0001). The proportion of male patients was significantly higher in 2020 than from 2016 to 2019 (*p* = 0.001).

**Conclusion:**

Public health measures against the COVID-19 pandemic likely decreased the prevalence of other respiratory tract infections. Further studies are needed to validate the effectiveness of each type of measure. Microbiological studies are also recommended, to better understand the effect of preventive measures.

## Introduction

Since the first case was recorded in Wuhan, China, the COVID-19 epidemic has spread to 222 countries and territories with more than 263 million cases, and it has claimed the lives of over 5 million persons [1]. This epidemic has affected the life, socio-economy, and health of individuals all over the world. It increases the medical burden even in countries with well-developed medical systems.

Vietnam faces a high risk of a severe COVID-19 outbreak because the country has a density population with nearly 100 million people and shares an approximately 1500 km border with China. As of December 1, 2021, 1,252,590 confirmed cases (992,052 recovered) and 25,448 deaths were reported [2].

Vietnam detected the two first cases on January 23, 2020 [3]. During the 5 weeks of the Lunar New Year, a total of 16 cases were recorded, all imported from Wuhan (https://ncov.vncdc.gov.vn/). COVID-19 imported in Vietnam from elsewhere than China started in early March with one case returned from London. Multiple effective measures have been applied to fight the COVID-19 pandemic in Vietnam such as: (i) early lockdown with series of flight cancelation, temporary closures of schools and all public places; (ii) a strong political commitment and prompt actions with a multi-sectoral response plan with the Ministry of Health playing a central role; (iii) blanket media coverage about COVID-19 prevention; (iv) intensive surveillance, case management, contact tracing for all COVID-19 newly confirmed cases and (v) large-scale health quarantine not only for patients but also for persons in close contact with cases [4]. From March 21, 2020, by compulsory quarantine for 14 days of all the persons who enter Vietnam, all the new cases were immediately isolated in health facilities.

These acts were based on the best evidence available at the time of the introduction of the virus in the country. The evidence consisted of global experience and previous pandemics [5, 6]. In some studies, social distancing reduced the spreading of seasonal influenza in workplaces [7]. The real effect of these broad restrictions on the spreading of diseases remains unclear, but it appeared to decrease the spreading of the novel coronavirus in China in 2020 [8]. The influenza season was shorter than normal, and the number of influenza cases was spectacularly decreased in 2020 [9–11], possibly because of the social restrictions and individual hygiene measures.

We aim to conduct this study to evaluate the impact of COVID-19 mitigation measures on the total number of consultations for respiratory tract infections (RTIs) and gastrointestinal infections (GIIs) among children of less than 16 years in Thai Binh Pediatric Hospital (TBPH), Vietnam during the year 2020.

## Materials and Methods

### Study Setting

Thai Binh is a province in the Red River Delta region of northern Vietnam with an area of 1542 km^2^. It is located in the southeast and about 110 km from Hanoi capital. In 2020, 38 COVID-19 adult patients have been confirmed by real-time polymerase chain reaction test in the Thai Binh province, of whom, 37 were repatriated persons. Only one autochthonous case was reported in the province. This case has had contact with a confirmed patient while visiting relatives at a hospital in Da Nang city, in central Vietnam.

TBPH is a central hospital in the province of Thai Binh. In 2019, this hospital had 565 beds spread across 15 different medical departments, including an outpatient department. Microbiological identification in the hospital laboratory uses common bacteriological cultures, serological assays and real-time polymerase chain reaction (PCR) for a limited number of pathogens [12].

### Study Design and Population

This is a retrospective review of consecutive consultations occurring in children aged 0–16 years admitted to the Thai Binh Pediatric Hospital from 1 January 2016 to 31 December 2020.

### Data Collection Methods and Instruments

All medical records of eligible subjects were collected from central computer of the hospital and analyzed by a team of three medical doctors. The eligibility criteria were consultation for health problems at the TBPH, documentation of the date and month of consultation, age, gender, and diagnosis according to the International Classification of Diseases (ICD10 criteria) (https://icd.who.int/browse10/2016/en#/). The medical data have been exported in Excel form. Statistical analyses were carried out using STATA version 15.1 (http://www.stata.com/). Categorical variables were presented as percentages. Chi^2^ test was used to compare proportion of RTIs and GIIs between two periods: “non-COVID-19 pandemic” (from 1 January 2016 to 31 December 2019) versus “COVID-19 pandemic” (from 1 January 2020 to 31 December 2020). Statistical test was considered significant when the *p*-value was < 0.05.

### Ethic

Ethical approval was obtained from the Ethics Committee of Thai Binh University of Medicine and Pharmacy (No. 303-HĐĐĐ).

## Results

**Fig. 1:**
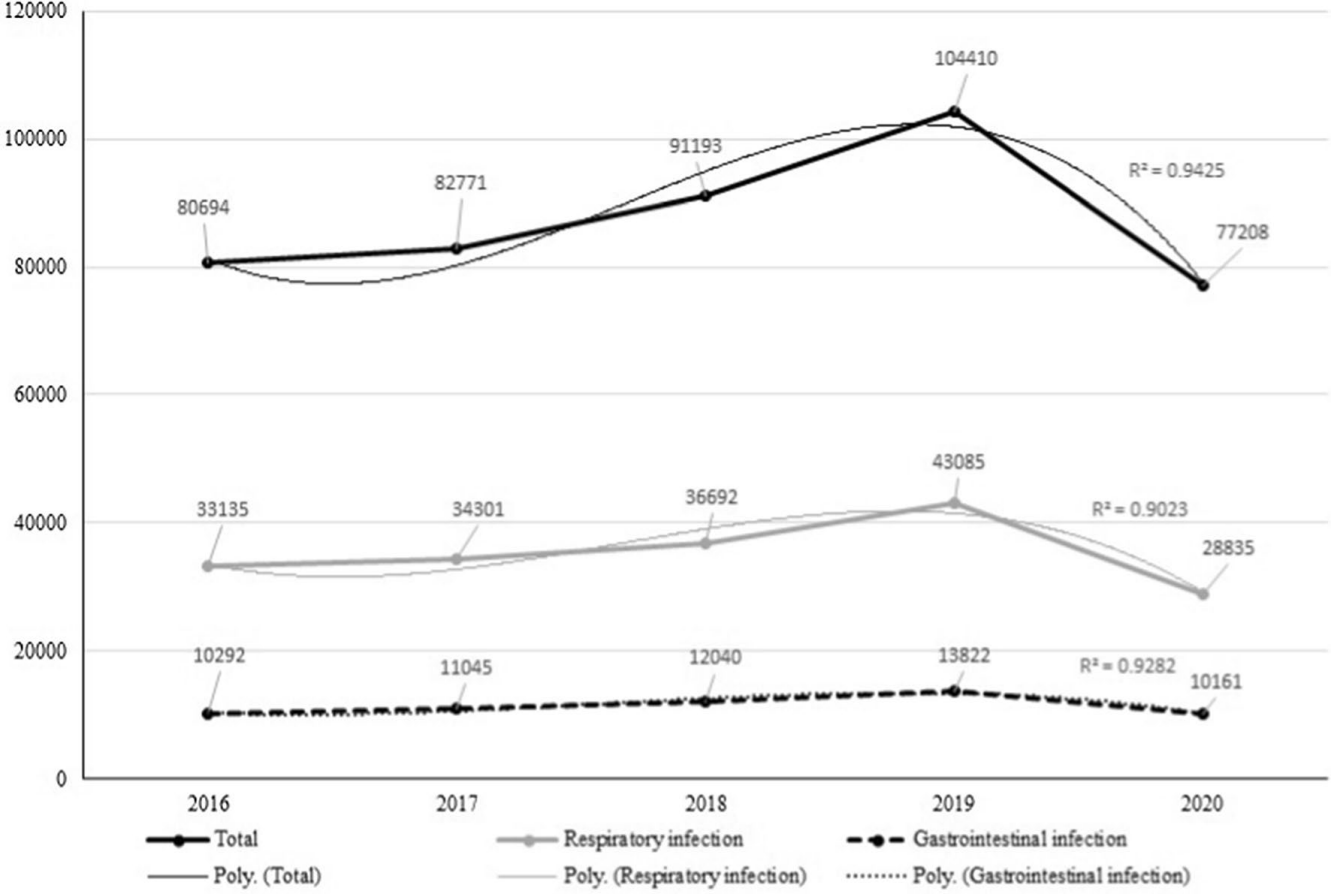
Change in number of visit in outpatient department, Thai Binh Pediatric Hospital from 2016 to 2020

During the period of study, 436,276 children of less than 16 years consulted at the outpatient department of TBPH (Fig. 1). We observed a gradual increase in the total number of outpatients from 2016 to 2019, including those consulting for RTIs and GIIs. By contrast, the total number of outpatients and the numbers of those consulting RTIs and GIIs dramatically decreased in 2020 (Fig. 1).

**Fig. 2:**
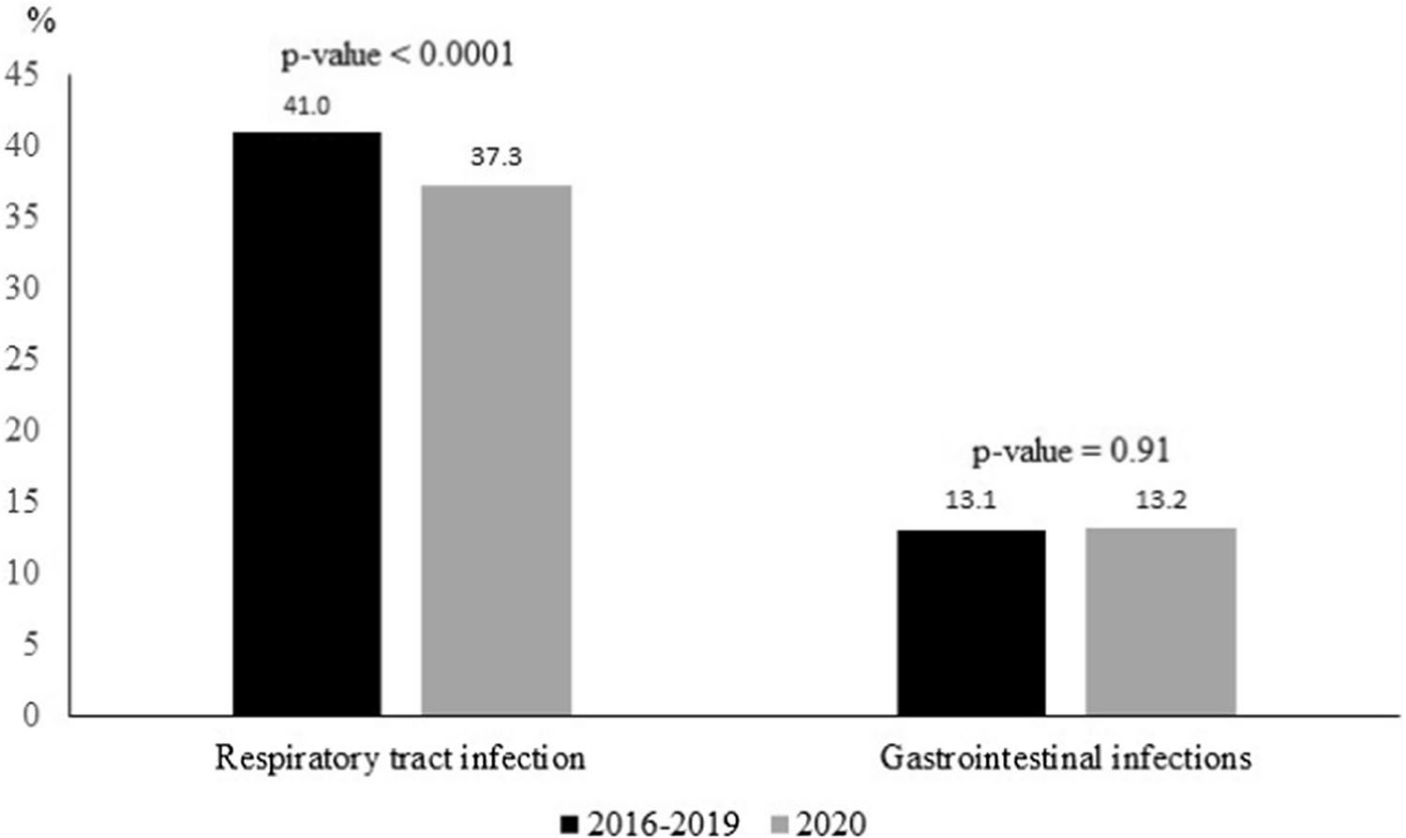
Relative proportions of respiratory tract infections and gastrointestinal infections during two periods: 2016–2019 versus 2020

Figure 2 shows the relative proportions of RTIs and GIIs during two periods: 2016–2019 versus 2020. A significant decrease of RTIs relative proportion was observed in 2020 (37.3%) when compared to 2016–2019 (41.0%) (*p* < 0.0001). By contrast, the relative proportion of GIIs (13.2% versus 13.1%, respectively) did not significantly vary with *p* = 0.91.

**Fig. 3:**
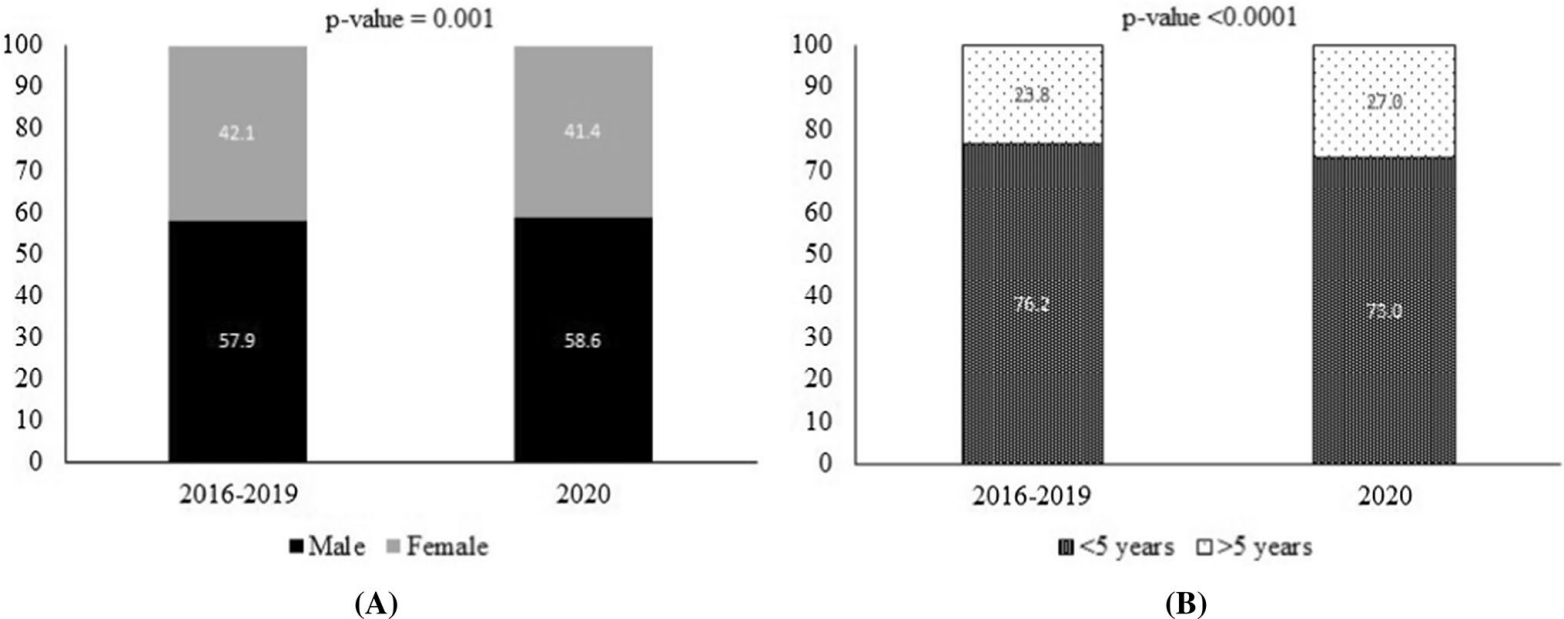
Changes in relative proportion of visits by gender (**A)** and age (**B**) of patients in Thai Binh Pediatric Hospital

Figure 3 shows the relative proportions of visits by gender and age during the period 2016–2019 versus 2020. The proportion of outpatients aged under 5 years was significantly lower in 2020 (73.0%) compared to previous years (76.2%) (*p* < 0.0001). The proportion of male patients was significantly higher in 2020 (58.6%) than from 2016 to 2019 (57.9%) (*p* = 0.001).

## Discussion

Several papers on epidemiological changes of RTIs were published to date [9, 13–23], but studies on GIIs during the COVID-19 pandemic remain scant [13, 24]. In a survey of Park et al., surveillance data from the South Korean National Respiratory Virus Sentinel Surveillance System showed a decreased number of hospitalized patients with acute viral respiratory infections in 2020. The peak of influenza-like illness during the COVID-19 epidemic was significantly lower than in 2016–2019 (48.8 cases per 1000 patients versus 86.2 cases per 1000 patients [14]. At Chongqing Health Center for Women and Children, China [15], before the COVID-19 pandemic, the pediatric outpatient volume showed an increasing trend from 2017 to 2019. Since December 2019, the number of outpatients and respiratory disease visits showed a significant gradual decrease [15]. Among 7107 hospitalized children (4600 in 2019 and 2507 in 2020) with lower RTIs in China, an unprecedented reduction of viral respiratory pathogens such as respiratory syncytial virus, adenovirus, influenza viruses was reported in 2020, despite of reopening of schools in June, 2020. However, the number of children infected with rhinovirus has increased significantly in 2020, especially after schools reopened [16].

A comparative study was conducted in Rochester, New York, United States, focused on assessing the impact of the pandemic on the incidence of acute otitis media and nasopharyngeal colonization among children in a primary care outpatient setting [17]. A total of 215 children were included in the pre-pandemic cohort and 144 in the pandemic cohort. A 1.8-fold less frequency of infectious disease visits was observed during the pandemic. Particularly, visits for acute otitis media and viral upper respiratory infections were nearly fourfold lower than the pre-pandemic cohort. In addition, the most frequent respiratory bacteria including *Streptococcus pneumoniae, Haemophilus influenzae* and *Moraxella catarrhalis* were less frequently isolated among ill children during the pandemic [17].

In a comparative study conducted in 994 general practitioners and 192 pediatrician practices in Germany, acute RTIs and GIIs were significantly decreased during the COVID-19 pandemic, compared with the non-pandemic period (− 62% for RTIs and − 40% for GIIS) [13].

A decrease in RTIs was seen following the implementation of preventive measure use against COVID-19, including mask-wearing recommendations among symptomatic individuals. Despite some inconsistent results [25, 26], the majority of studies demonstrated that facemask is an effective preventive measure against respiratory tract infections in various contexts [27]. In fact, the effectiveness of facemask and hand hygiene to prevent the spread of viral RTIs has been proven in previous studies [28, 29]. When patients with RTIs used facemask, it helps to reduce the spread of contaminated droplets during coughing and sneezing. In Singapore, a decrease in respiratory infections due to rhinovirus at three major public hospitals was seen during the lockdown, and after that, this virus rebounded earlier than others. Mandatory mask wearing did not appear to prevent it [18]. Rhinovirus is a small, non-enveloped hydrophilic virus which is stronger than others, and has a relatively greater propensity for contact transmission [18]. Therefore, rhinovirus infection easily re-starts after schools reopen [30]. In this study, our results also showed that RTIs, in general, tended to decrease during the COVID-19 pandemic, but not in children over 5 years old.

Our study has some limitations. First, it is based only on clinical diagnosis without microbiological confirmation. Furthermore, the results of this monocentric study cannot be extrapolated to the whole pediatric population of Vietnam.

## Conclusion

In conclusion, public health measure against the COVID-19 pandemic likely decreased the prevalence of RTIs and GIIs. Further studies are needed to validate the effectiveness of each type of measure. On the other hand, microbiological studies are also recommended, to better understand the effect of preventive measures.

## Data Availability

The data that support the findings of this study are available from the corresponding author, [PG], upon reasonable request.

## Abbreviations

COVID-19: Coronavirus disease 2019
GIIs: Gastrointestinal infections
RTIs: Respiratory tract infections
TBPH: Thai Binh pediatric hospital
